# Implementation of a neonatal pain management module in the computerized physician order entry system

**DOI:** 10.1186/2110-5820-2-38

**Published:** 2012-08-22

**Authors:** Nathalie Mazars, Christophe Milési, Ricardo Carbajal, Renault Mesnage, Clémentine Combes, Aline Rideau Batista Novais, Gilles Cambonie

**Affiliations:** 1 Neonatology Department, Arnaud de Villeneuve Hospital, CHU Montpellier, Montpellier, F-34000, France; 2 Pediatric Emergency Unit, Armand Trousseau Hospital, Assistance Publique-Hôpitaux de Paris, Paris, F-75000, France; 3 INSERM U953 Pierre and Marie Curie University, Paris, France; 4 Neonatology and Intensive Care Unit, Montpellier University Hospital Centre, Arnaud de Villeneuve Hospital, 371 Avenue du Doyen G Giraud, 34295, Montpellier, Cedex 5, France

**Keywords:** Analgesia, Computer-assisted instruction, Newborn, Pain management, Sedation

## Abstract

**Background:**

Despite the recommended guidelines, the neonatal management of pain and discomfort often remains inadequate. The purpose of the present study was to determine whether adding a pain and discomfort module to a computerized physician order entry (CPOE) system would improve pain and discomfort evaluation in premature newborns under invasive ventilation.

**Methods:**

All newborns <37 weeks gestational age (GA) and requiring invasive ventilation were included in a prospective study during two 6-month periods: before and after the inclusion of the pain and discomfort evaluation module. The main outcome measure was the percentage of patients having at least one assessment of pain and discomfort per day of invasive ventilation using the COMFORT scale.

**Results:**

A total of 122 patients were included: 53 before and 69 after the incorporation of the module. The mean age was 30 (3) weeks GA. After the module was included, the percentage of patients who benefited from at least one pain and discomfort assessment per day increased from 64% to 88% (*p* < 0.01), and the mean number (SD) of scores recorded per day increased from 1 (1) to 3 (1) (*p* < 0.01). When the score was not within the established range, the nursing staff adapted analgesia/sedation doses more frequently after module inclusion (53% vs. 34%, *p* < 0.001). Despite higher mean doses of midazolam after module introduction [47 (45) vs. 31 (18) μg/kg/hr, *p* < 0.05], the durations of invasive ventilation and hospital stay, and the number of nosocomial infections, were not significantly modified.

**Conclusions:**

Adding a pain and discomfort tool to the CPOE system was a simple and effective way to improve the systematic evaluation of premature newborns who required ventilatory assistance.

## Background

Premature newborns hospitalized in intensive care undergo many painful medical acts, with some studies signaling an average of 15 such acts per day [1,2]. Managing the pain and discomfort (PAD) of these infants is a therapeutic priority because of the immediate consequences to the infants’ stability [3] and the long-term repercussions on neuroendocrine development and the capacity to manage stress from nociceptive stimuli [4-6].

Although the message that relieving and preventing PAD in newborns has been widely disseminated to all concerned medical staff, PAD management remains inadequate [7,8]. In 2006, a rigorous analysis of the literature suggested that these practices could be improved by establishing precise objectives, including the systematic evaluation of PAD and the development and formalization of protocols for its management [9,10].

On our unit, many actions have been initiated since 2002: the creation of a “pain group,” biannual training courses about pain evaluation and treatment, placement of COMFORT scales in front of each incubator, and the construction of a pain management protocol available in paper and electronic form. However, during a recent audit on PAD management in our NICU, we found that the most important objective we needed to reach was the systematization of PAD evaluation. We observed that only two thirds of the neonates under invasive ventilatory assistance had benefited from at least one formalized evaluation per day; that is, with the use of a checklist. This situation prompted us to develop a software module to specifically manage PAD, which was included in our daily-use computerized physician order entry (CPOE) system. We chose this tool for two reasons. First, we assumed that the module would encourage physicians to become more directly involved in pain management, because they would have to validate an algorithm daily to evaluate PAD and adapt analgesic/sedative treatment, based on the unit protocol. Second, we assumed that this tool would encourage the nurses to score the pain objectively, because by doing so they would be able to adjust the analgesic/sedative doses on the basis of their evaluation.

The purpose of this study was to evaluate whether the inclusion of a PAD evaluation module in the NICU CPOE system would be an effective strategy to improve PAD evaluation in premature newborns requiring invasive ventilation.

## Methods

This prospective, before-after study was conducted in the 12-bed NICU of a tertiary care university hospital in Montpellier, France. The ratio of pediatric nurses to infants is 1 to 2 and the shift rotation is every 12 hours.

### Local protocol for managing pain and discomfort

Since 2002, our NICU staff has followed the protocol that we collectively developed for managing pain and discomfort; this protocol can be consulted in paper form in the NICU protocol binder. A printed version of the COMFORT scale for evaluating pain and discomfort is included in all patient charts, and all medical and paramedical personnel receive training in pain and discomfort evaluation and management twice a year.

#### COMFORT scale

The COMFORT scale assesses several components of pain and discomfort and has been validated in premature newborns under ventilation [11,12]. The components are essentially observable behaviors and variations in physiological criteria. The sum of the different items ranges from 8 to 40. Different cutoff or range values for the COMFORT scale have been proposed or established to describe the infant’s status in terms of pain and discomfort [13,14]. On our unit, we follow the recommendations of the Centre National de Ressources de lutte contre la douleur (CNED; National Center for Resources to Combat Pain; http://www.pediadol.org/IMG/pdf/COMFORT.pdf). Newborns are assumed to be “pain-free and comfortable” with scores between 18 and 23. They are considered to be “in pain or uncomfortable” with scores between 24 and 40, and “excessively sedated” with scores between 8 and 17. The interrater reliability of the COMFORT scale was verified when the scale was introduced on the NICU in 2002, but it was not repeated before beginning the study.

#### NICU protocol

The protocol remained the same and was presented to all staff before each phase of the study. All newborns under invasive ventilation received an opioid (sufentanil, starting dose: 0.1 μg/kg/hr) in combination with a benzodiazepine (midazolam, starting dose: 30 μg/kg/hr). The efficacy was evaluated every 8 hours by a pediatric nurse using the COMFORT scale and every 4 hours if a painful procedure had been performed. The objective was a score between 18 and 24. For a score >24, the first step was to determine whether there was a specific cause, such as product perfusion or respiratory obstruction, and to improve the environmental conditions, notably by changing the baby’s position and the respirator settings if necessary. Then, if the excessively high COMFORT score was judged to be directly pain-related—for example, following a painful medical act—a bolus corresponding to an hourly dose of sufentanil was administered over 10 min and the hourly dose was increased by 20% if the score remained elevated after two boluses. If the excessively high COMFORT score was judged to be the result of discomfort—for example, a hyperalert newborn without a recent medical act or stimulation who seemed to be struggling against the respirator—the hourly dose of midazolam was increased by 20%. If the score was <18, we began reducing the hourly dose of analgesia/sedation by 20% over 8 hours, usually beginning with the midazolam. All the patients also were managed according to the Neonatal Individualized Development Care and Assessment Program used on our NICU since 2004 [15].

### Computerized pain evaluation module

**Figure 1:**
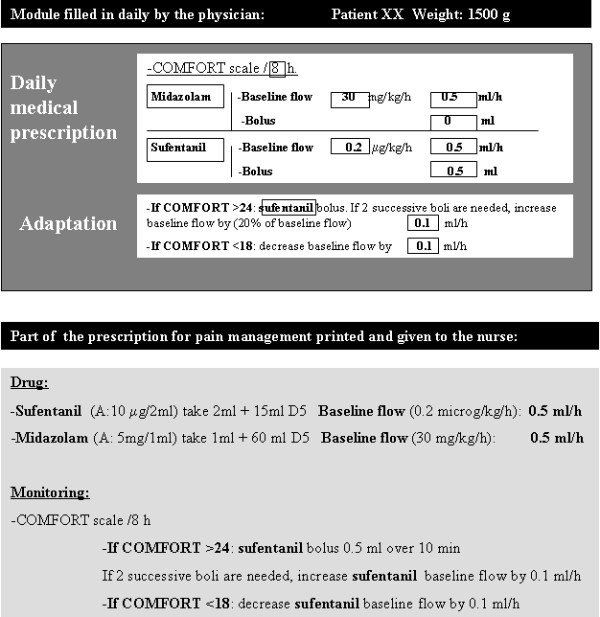
**Example of the specific window for pain and discomfort evaluation and management.** The window was activated during the entry of computerized physician orders and the retranscription printed out and given to the nurse. The physician could modify the frequency of evaluation with the COMFORT scale, and the baseline dosage and bolus dosages of analgesic and/or sedative.

A CPOE system was introduced on our unit in 2001 to limit the risk of medical errors [16]. The module was added to the CPOE system in such a way that, each time the software was run, a specific window opened first. Every day, this window prompted the physician to order COMFORT scale assessment at a modifiable frequency and the adaptation of sufentanil and/or midazolam dosages in line with the protocol. The physician then discussed the situation with the nurse in charge of the infant and sometimes modified the orders and validated them. The orders were then printed out, with the individualized PAD management algorithm added to the other medication and surveillance orders for the day (Figure 1). All of the therapeutic adaptations were thus carried out by the nurses according to the algorithm. The physician was notified if the increase or decrease in dosages or bolus administration did not lead to normalization of the COMFORT score in the ensuing 30 minutes.

### Population

All premature newborns with GA <37 weeks and requiring invasive mechanical ventilation were eligible for study inclusion. The noninclusion criteria were the following: a decision to limit or stop life-sustaining therapy and the use of curare during invasive ventilation.

### Study design

The patients were prospectively enrolled in the study during two 6-month periods separated by the introduction of the computerized module for PAD evaluation and the adaptation of analgesic/sedative dosages based on the score. Two groups of patients were compared.

#### Control group

The control group was recruited during the period before the introduction of the PAD module, between September 2008 and February 2009. The orders to evaluate PAD and then modify the medication dosages on the basis of the score followed the protocol established in our unit; this protocol was available to all staff but could be adapted by each prescribing physician.

#### Intervention group

This group of patients was recruited after the introduction of the PAD evaluation module on March 1, 2009. It thus included the patients in the immediate postintervention period from March to August 2009. During this period, the protocol for PAD management remained the same on the NICU, notably regarding the nurses, who were able to adapt the analgesic/sedation dosages based on the COMFORT scores. The only modification in this period was the systematization of PAD assessment as part of the written orders given to them.

### Criteria for weaning from invasive ventilation

Newborns were extubated when they presented all of the following criteria: 1) clinical stability and spontaneous autonomous ventilation on the endotracheal tube verified during routine care, such as changing diapers or modifying position; 2) positive expiratory pressure <4 cm H_2_O and maximal inspiratory pressure <20 cm H_2_O and FiO_2_ <0.4; and 3) pH >7.25 and SpO_2_ >85% [17]. Infants <28 weeks were systematically placed on nasal continuous airway pressure after the weaning from invasive ventilation.

### Prevention of withdrawal syndrome

Once the decision to wean the infant from invasive ventilation was made, the NICU protocol called for a daily 20% reduction in the drug dosage when analgesic/sedatives had been given for more than 5 days and 10% when the drugs had been given for more than 10 days. In addition, withdrawal syndrome was systematically evaluated by the neonatal abstinence score of Finnegan [18].

### Data collection

The nurse in charge of the infant noted the exact time of PAD assessment and the therapeutic response (name of the drug, dose, and adaptation) in the patient chart. The main outcome measure was the percentage of patients having at least one evaluation per day of ventilation using the COMFORT scale, i.e., the ratio of the number of patients having at least one evaluation per day to the number of patients ventilated. During the ventilation period, if one of the daily nursing forms for hourly surveillance did not have a COMFORT score recorded, the infant was not included in the numerator but was included in the denominator. We arbitrarily chose the compliance rate of only one evaluation per day of ventilation in the absence of firm recommendations on the rate of evaluation in these patients.

The secondary outcome measures were the number of scores prescribed and assessed per day of ventilation, the mean COMFORT score, the mean dosages of analgesics and sedatives, the duration of ventilation, the duration of hospitalization, and the number of nosocomial infections.

### Statistical analysis

To calculate the number of subjects needed, previous data collections suggested that the prevalence of at least a daily evaluation of the COMFORT score in the control group would be 65%. Given an alpha risk of 5% and a power of 80%, 50 patients per group were needed to show an increase in this prevalence, from 65% before module inclusion to 90% after, corresponding to a 38% improvement in scoring. Considering the mean number of premature infants admitted to our NICU for invasive ventilation, we chose a 6-month inclusion period for both the pre- and postintervention phases.

Summary statistics are given as means (SD) for continuous variables and as numbers (percentages) for categorical variables. Categorical variables were compared with the chi-squared test or Fisher’s exact test when necessary. For continuous variables, group comparisons were made with Student’s *t* test or the Mann–Whitney when distributions were not normal or the variances were unequal. Statistical significance was set at 5% for all tests. Statistical analysis was conducted with the SAS software package, version 9 (SAS Institute, Cary, NC).

### Ethical considerations

The parents of all children gave written, informed consent to care. The procedures described herein are all part of routine care on our unit and did not require approval of the institutional review board, in line with French law.

## Results

### Population

From September 2008 to August 2009, 349 newborns <37 weeks GA were admitted to the NICU; 214 of them did not require invasive mechanical ventilation. Of the 135 who required invasive ventilatory assistance, 10 were excluded from analysis following the decision to limit or stop life-sustaining therapy and 3 because of missing data. No patient was treated with curare. Thus, 122 patients were included: 53 in the control group and 69 in the intervention group.

### Patient characteristics (Table 1)

**Table 1:** Clinical characteristics of patients, before (Control) and after (Intervention) inclusion of the module for evaluation and management of pain and discomfort

	**Control**	**Intervention**	
	**n = 53**	**n = 69**	***p***
Gestational age, wk	29 (3)	30 (3)	0.07
Birth weight, g	1380 (620)	1440 (610)	0.6
CRIB score	7 (4)	6 (4)	0.17
Small for gestational age, n (%)	10 (19)	10 (14)	0.79
Surfactant use, n (%)	45 (81)	58 (84)	0.98
Pneumothorax, n(%)	4 (7)	1 (1)	0.13
Grade 3–4 IVH, n (%)	6 (11)	10 (14)	0.93
Periventricular leukomalacia, n (%)	2 (4)	6 (9)	0.28
Necrotizing enterocolitis, n (%)	2 (4)	6 (9)	0.68
Died during study period, n (%)	5 (9)	8 (11)	0.49

Data are means (SD) or numbers (n) and percentages. IVH: Intraventricular hemorrhage.

Birth weight, gestational age and CRIB score were comparable between the two groups at the time of admission.

### Evaluation and management of PAD before and after the computerized pain module inclusion (Tables 2 and 3)

**Table 2:** Changes in pain and discomfort (PAD) evaluation and management and other parameters of NICU hospitalization, before (Control) and after (Intervention) inclusion of the PAD module

	**Control**	**Intervention**	
	**n = 53**	**n = 69**	***p***
Infants whose pain was assessed daily, n (%)	34 (64)	61 (88)	0.002
Number of assessments prescribed daily	1 (2)	3 (2)	<0.01
Number of assessments performed daily	1 (1)	3 (1)	<0.01
COMFORT score			
-Number of scores	813	1380	
-Mean value	20 (3)	19 (3)	0.07
-Scores outside the target range, n (%)	182 (22)	310 (22)	0.96
Absence of drug adaptation by the nurse			
-For scores outside the target range, n (%)	119 (15)	145 (10)	<0.01
-For scores >24, n (%)	11 (2)	6 (0.5)	0.04
-For scores <18, n (%)	108 (13)	139 (9.5)	0.03
Nosocomial infection, n (%)	13 (25)	20 (29)	0.61
Length of invasive ventilation, days	7 (9)	8 (11)	0.59
Length of stay in the NICU, days	23 (17)	20 (20)	0.38

Data are means (SD) or numbers (n) and percentages.

**Table 3:** Analgesic and sedative drugs and their potential side effects, before (Control) and after (Intervention) inclusion of the pain and discomfort module

	**Control**	**Intervention**	
	**n = 53**	**n = 69**	***p***
Patients with sedatives or analgesics, n (%)	35 (66)	52 (76)	0.23
Sufentanil, μg/kg/hr	0.16 (0.1)	0.16 (0.08)	1
Midazolam, μg/kg/hr	31 (18)	47 (45)	0.016
Fluid bolus and/or vasoactive drugs, n (%)	10 (19)	13 (19)	1
Preventive treatment of WS, n (%)	20 (38)	20 (29)	0.46

Data are means (SD) or numbers (n) and percentages. WS, withdrawal syndrome.

The percentage of patients having at least one evaluation per day of ventilation using the COMFORT scale increased from 64% before module inclusion to 88% after inclusion (*p* = 0.002).

**Figure 2:**
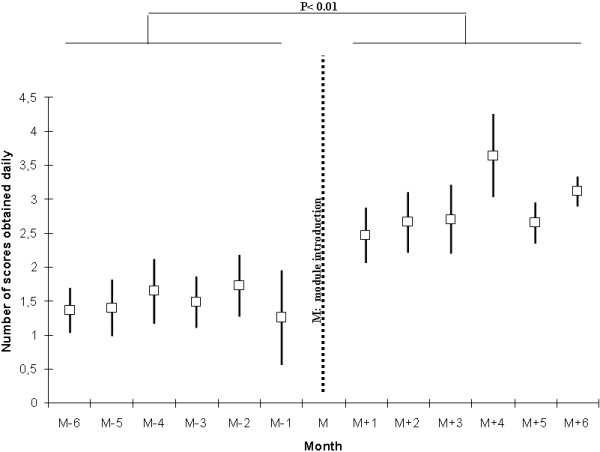
**Number of individual daily COMFORT scores during mechanical invasive ventilation, before and after module inclusion.** Data are means and SD.

The mean number (SD) of COMFORT scores ordered per newborn and per day of ventilation also increased from 1 (2) before the intervention to 3 (2) after intervention (*p* < 0.01, Figure 2).

The COMFORT scores fell outside the target range 182 times of the 813 scores collected before the intervention (22%) and 310 times of the 1,380 scores after intervention (also 22%).

When the target range of 18 to 24 was not reached, the nursing staff more often adjusted the analgesic/sedative dosages after intervention (53% vs. 34%, *p* < 0.001).

The percentage of patients who received analgesia/sedation during invasive ventilation was comparable in the two groups: 66% vs. 76%, *p* = 0.23. The mean hourly dose of sufentanil was not modified after the intervention, whereas midazolam was higher. This change was not associated with hypotension significant enough to require fluid boluses and/or vasoactive drugs. A strategy to prevent withdrawal syndrome was implemented for 20 infants in each study period, and no withdrawal syndrome was observed over the entire study (Table 3).

### Pain module inclusion and changes in the parameters of NICU hospitalization

The duration of invasive mechanical ventilation, the duration of the NICU stay, and the rates of nosocomial infection and mortality remained comparable before and after intervention (Table 2).

## Discussion

This study showed that adding a PAD module to a NICU CPOE system improved both PAD evaluation and analgesia/sedation dose adaptation on the basis of the scores. The midazolam dosage was increased after module inclusion, whereas the durations of ventilation and NICU stay were not affected.

The evaluation of PAD is mandatory in France and is part of the nursing role (Decree of competence No. 2004–802 of July 29, 2004, regarding professional nursing acts and the exercise of the nursing profession). Yet despite this, PAD evaluation remains inadequate on intensive care units—whether neonatal, pediatric, or adult—with the prevalence of patients evaluated ranging from 40% to 70% [7,19,20]. Including a specific module in our CPOE system thus seemed to be a potential means to improve the evaluation rate for our nursing staff. For the physicians, a daily suggestion to validate an algorithm for adapting analgesia/sedation dosages on the basis of COMFORT scores was expected to lead to systematic discussions with the nurses on the PAD of the individual patient. For the nurses, the immediate association of a score with a therapeutic response was expected to reinforce the importance of regular PAD evaluation [21,22]. Nurses feel supported in performing these evaluations when they know that the physicians are strongly involved [23]. The inclusion of the module improved drug adaptation by the nurses, whether the scores were high or low. However, in both periods, the nurses were less inclined to act when scores were low. This result points to the need to transmit a balanced message on PAD management by also emphasizing the risks associated with excessive sedation and analgesia.

The durations of invasive ventilation and NICU hospitalization and the rate of nosocomial infection remained comparable before and after the intervention. The literature has shown conflicting results regarding the impact of an analgesia/sedation algorithm on the duration of invasive ventilation [24,25]. Only certain studies on adult patients have demonstrated a shorter ventilation period [21,23,26-28] or a benefit in terms of the pneumonia associated with mechanical ventilation [26]. However, the protocols for adult ICUs include daily interruption of analgesia/sedation to determine whether the patient can be extubated [28,29]. This strategy, which may in itself reduce the time of ventilation, is rarely used in pediatrics [30] and was not employed on our unit. In any case, the small number of subjects in our study did not permit any conclusions to be drawn regarding this secondary outcome. Moreover, the algorithm for evaluation and management of PAD was the same during the two study periods. We studied only the systematization of this algorithm and not its implementation.

We chose the COMFORT scale over other multidimensional PAD scales, because it has been validated for use in newborns to assess objectively the adequacy of analgesia/sedation and it is widely used [13,19,31]. However, as opposed to the situation for adults, no scale is currently able to distinguish pain from discomfort in newborns [32,33]. This lack of specificity in the COMFORT scale implies a certain degree of subjectivity in the interpretation of scores, and frequent discussions between physicians and nurses are needed to determine the best therapeutic choice as pain and discomfort are managed differently. As noted in the *Methods* section, we optimized the environmental conditions before proceeding to a therapeutic adaptation of either midazolam if the infant was assessed as “uncomfortable or agitated” or sufentanil if assessed as “hyperalgesic.” It was noteworthy that the more systematized evaluation of PAD, following the addition of the module, led to an increase in the prescription of sedatives and not analgesics. One explanation is that our NICU staff has become very sensitized to pain management since 2002. When a newborn is exposed to a situation likely to generate pain, evaluation and management of the pain is nearly systematic. In contrast, our results indicated that situations of discomfort were underestimated by the nursing staff and that multidimensional scales might help to better sensitize them to discomfort and its management, through medication or other means.

Whether the use of sedation and analgesia is recommended during invasive ventilation in newborns, the use of midazolam remains controversial [34]. Nevertheless, this drug is widely used on French NICUs, and no adverse effect was found at the age of 5 years in a large cohort of premature infants exposed to extended sedation/analgesia compared with an unexposed group [35].

The limitations of the study include the pre-post design. The CPOE system had been in use on our unit before the study began, and thus randomization with and without a computerized tool was not feasible. We therefore used a simple before–after design for the pain and discomfort module. However, this method is open to criticism and did not allow us to affirm that our results were the exclusive consequence of module introduction. Alternative explanations include unnoticed changes in the patient characteristics at inclusion or in the management protocols on the NICU. The nursing staff turnover was 6% during the study, and we also cannot exclude that some of the “new” nurses had undertaken personal training on pain. Indeed, these nurses were generally young and perhaps more sensitized to pain management. With a short period of observation, only 6 months after module introduction, we cannot assume that the observed changes were sustained over time. The limited number of infants, the monocentric nature of the study, and the specificities of our “homemade” module also are limitations for extrapolating our data to other NICUs. Nevertheless, we believe that our results will serve to encourage reflection on how to improve PAD evaluation in critically ill newborns.

## Conclusions

The addition of a PAD module to an NICU CPOE system seems to have been an effective means to improve the systematic evaluation of PAD in premature newborns under invasive ventilation. Further studies at a bigger scale are now needed to validate this type of resource in the daily management of PAD in neonatology.

## Abbreviations

CPOE: computerized physician order entry; GA: gestational age; NICU: neonatal intensive care unit; PAD: pain and discomfort.

## Competing interests

The authors declare that they have no competing interests.

## Authors’ contributions

All authors are legitimate and have actively contributed to the work as follows: NM and CM: experts in our team for pain and discomfort management: study design, data collection, and manuscript preparation. RC: international expert in pain assessment and management: technical advice about the study design, manuscript preparation, review, and correction. RM: developed the computerized physician order entry system and the pain and discomfort module on our unit. CC and ARBN: contribution to the methodological part of the study design, monitoring, management of the data, statistical analysis of the data, and reviewing the manuscript. GC: study design and interpretation of the data, wrote the paper, and is the corresponding author. All authors read and approve the final manuscript.

## Authors’ information

This work was carried out in the Neonatal Intensive Care Unit (NICU) of Arnaud de Villeneuve Hospital, CHU Montpellier, F-34000 France.
